# You can lead a horse to the water….

**DOI:** 10.4103/0019-5545.37660

**Published:** 2007

**Authors:** G. Swaminath

**Affiliations:** Department of Psychiatry, Dr. B. R. Ambedkar Medical College, Kadugondanahalli, Bangalore - 560045, India

## Prescription adherence - on whom is the onus?

One of the most frustrating experiences faced by a physician is having a recovered patient return with recurrence of symptoms because the patient, his family or both together had decided to discontinue the treatment. The disappointment is compounded if the patient despite a prediction of poor outcome, had shown excellent recovery. Similar dissatisfaction is faced by psychiatrists who find their chronic psychotically ill relapse after discontinuing medication after having shown good improvement and are now unmanageable and violent. The annoyance of the treating doctor at the beseeching relatives who return sheepishly, guiltily, helplessly or even arrogantly at times and implore immediate remedial measures for the patient, is understandable.

This raises an important issue of poor compliance in psychiatric and other chronic disorders. Our society relies upon medications to improve the clinical outcomes of morbidity and mortality and to reduce or prevent hospitalization and to improve quality of life. However, many patients do not realize the full potential benefits of prescription therapies, resulting in their failure to take some or all of the medications prescribed by their physician. The consequence is a decrease in quality-of-life improvement derived from medicines and unnecessary increases in avoidable healthcare cost.[1]

## Compliance, concordance, adherence and persistence

There are various terms used to describe such patient behaviour. The commonest and most traditional term is *Compliance*, which suggests a passive approach where the patient faithfully (and often without question) follows the advice and directions of the healthcare provider.[1] It denotes a paternalistic point of view of the practitioner- patient relationship. However the term has negative connotations and suggests yielding, complaisance and submission.[2] Inherent to all the various definitions of compliance is the assumption that medical advice is good for the patient or that rational patient behaviour means following medical advice precisely.[3]

However there could be something morally and psychologically flawed in the concept of compliance. Non-compliance can be defined as a person's informed decision not to adhere to a therapeutic regimen.[4] Compliance is the extent to which an individual chooses a behaviour that coincides with a clinical prescription, whereas another term suggested by the Royal Pharmaceutical Society of Great Britain, *Concordance* is the patient's considered choice.[5] Concordance indicates the extent to which what the patient thinks about what is asked from him matches what the health care-giver thinks the patient actually does.[2] Another term, *Adherence* has also been proposed as an alternative to compliance which reduces attribution of greater power to the doctor in the doctor-patient relationship. Adherence is a collaboration between the patient and healthcare provider regarding health-related decisions. Utilizing this terminology (adherence) with the patient assists in fostering ownership and the continuation of treatment decisions by the patient. The term adherence incorporates the broader notions of concordance, cooperation and partnership.[2] Another term used is *Persistence*, which is the ability to see a prescribed therapy through to its intended completion, in other words, beginning therapy and continuing to use the medication and refill the prescription until instructed otherwise.[1]

All this suggests that resistance to taking drugs is profound and pervasive. What ever be the means of defining the problem this issue has become one of the serious issues governing the doctor patient relationship. Nonadherence to prescribed treatment regimens jeopardizes the outcome of treatment for every medical and psychiatric condition and has been called “America's other drug problem”.[6] This is universally true.

The process of seeking, receiving and following treatment and advice has many stages and many opportunities for non-compliance. Different types of non-compliance include:[7] delay in seeking care (population at risk), non-participation in health programmes (screening), breaking of appointments (follow-up), failure to follow doctors' instructions (treatment).[8] Further types can be distinguished: receiving a prescription, but not having it made up at a pharmacy (primary non-adherence), taking an incorrect dose, taking the medication at wrong times, forgetting one or more doses of the medication, stopping the treatment too soon, either by ceasing to take the medication sooner than the doctor recommended or failing to obtain a repeat prescription (secondary non-adherence).[9]

## Non adherence and psychiatric illnesses

Non adherence in psychiatric illnesses is influenced by the triad of medication, patient and the psychiatrist patient partnership. The numbers of doses and concurrent medication, adverse effects as well as long term medication as prophylaxis are medication related factors.[1]

Psychiatric disease itself is a significant barrier to adherence and many affected patients have complicating co-morbid conditions (like substance abuse and homelessness). Denial of illness and lack of insight compound the problems in psychiatric patients.[10] These are major patient related factors.

However the most important factors fall under the rubric of Healthcare-provider-related factors and include a) a poor healthcare provider-patient relationship, b) poor healthcare-provider communication skills (contributing to lack of patient knowledge or understanding of the treatment regimen), c) disparity between the health beliefs of the healthcare provider and those of the patient and finally d) no positive reinforcement from the healthcare provider.[1] These issues need to be addressed in the doctor patient relationship to improve adherence.

It is important for the practitioner to understand that managing adherence is a continuous and dynamic process. Therefore, a patient's ability to adhere to therapy should be reassessed on a regular basis. Even the most conscientious of individuals may not be able to follow the directions and suggestions of their clinician exactly. The degree of compliance that is “good enough” will change depending on the medical condition and the consequences that go along with non treatment of the disorder.[1]

## Assessing and Motivating Health Behavior Change

The following are the various suggestions to assess and motivate patients to adhere to treatment recommendations:[9,11]

1. Educate the patient about the goals of therapy, the illness, the benefits of treatment and the importance of adherence to the regimen.
2. Anticipate and treat side effects.
3. Reduce dosage frequency and number of pills and memory aids (e.g, phone reminders, medication timers).
4. Recruit the patient's family and friends for support and to reinforce adherence.
5. Monitor adherence and intensify management in periods of low adherence.
6. Reinforce adherence during planned discharge and after care environment.
7. Consider the impact of new diagnoses (e.g, depression, recurrent chemical dependence) and include adherence intervention in the management plan. Concurrent substance abuse is a very important reason for non adherence of medication in the severely psychiatrically ill. Patients might choose alcohol over prescription medication when told not to consume alcohol. Patients avoid medications as some of them counteract the effects of the alcohol or drugs (so the person cannot experience their desired high).
8. Use of a “patient-centred” approach, recognise the patient as a consumer of services.
9. Of the interventions to improve adherence those more likely to be successful focused on attitudes and beliefs about medications as opposed to those focusing only on knowledge. Interventions that focused primarily on medication adherence were more likely to be successful than counsel with a broad educational focus
10. Expect and plan for noncompliance and non-adherence; don't wait for it to occur. Be vigilant and check repeatedly.

## Motivational Interviewing

The change from non-adherence to adherence is gradual and deliberate. Prochaska and Diclemente's[12] trans-theoretical model of behavior change identifies 5 stages of change: pre-contemplation, contemplation, preparation, action and maintenance These self-descriptive stages point to the individual's willingness to think about, make and maintain change.

Motivational interviewing (MI) is a method of interacting with patients to assess their readiness for change and to facilitate movement from one stage to the next. This is done by addressing a patient's ambivalence about change and illuminating their personal pros and cons of change. MI has been used in many health settings and been shown to be successful in promoting smoking cessation, weight loss, increased exercise[13] and reduced alcohol/drug use.[14] Originating in the addiction field, MI has been successfully adapted for brief medical consultations.[14]

The basic components of MI include building rapport, avoiding resistance, selecting an agenda, assessing readiness to change, addressing ambivalence, determining the level of importance of the issue and evaluating the level of the patient's confidence in his or her ability to change.[13] The tone and demeanor adopted when using MI is nonjudgmental, empathic and encouraging. A main objective of MI, in addition to assessing a patient's readiness to change, is to illuminate the discrepancy between the patient's desired goal and their current health behavior[14] and help patients consciously realize their readiness to change and their ambivalence about change.

Emmons and Rollnick[15] suggest that understanding change from the patient's point of view is an important aspect of the spirit of MI. Two particularly relevant points are (1) readiness to change is a not a client trait but a fluctuating product of interpersonal interactions and (2) the desire to change should be “elicited,” not “imposed.” It is important to recognize that rational arguments are not effective in resolving ambivalence.

Research results have shown that providing appropriately timed information to an individual is an important consideration when promoting or supporting health behavior changes. An individual may cycle back and forth through the stages several times in the process of changing behaviors. The “decisional balance” shifting an individual to make change occurs when they decide that the cons outweigh the pros of maintaining a behavior. It is at this point that an individual is open to information about carrying out a change. Providing information about behavior changes before an individual is ready to consider a change is like forcing a horse to drink water.[14]

Bedell *et al*,[16] discuss the power of language and “healing words.” It is noted that use of jargon and potentially disturbing metaphors is not uncommon in physician-patient communication. The reason for not using clear, compassionate language are time pressures and a desire to present a “definitive solution”, an impulse to “convey a sense of urgency” and the lack of awareness of what is actually said. Bedell and colleagues suggest physicians increase efforts to use “everyday language” that is “precise” and “clarifies the patient's priorities.” They further suggest including family members if a patient is highly anxious and reinforcing verbal messages with nonverbal behavior.

We therefore need to look beyond patient education if we are to reduce non-adherence to treatment and perhaps education of professionals should be the next target. Postlethwaite *et al* in a letter to the BMJ on this subject quote Wolff *et al*, who have said, “With few exceptions non-compliance is regarded as the patient's fault rather than exploring organizational or interactive reasons for non-compliance.”[17]

## Improving chances of the horse drinking

Adherence to therapy is essential to an optimal clinical outcome in patients with chronic diseases, especially psychiatric illness. These patients are at increased risk for poor adherence to treatment and thus for poor outcomes.

There are specific patient, disease, medication and treatment characteristics that negatively affect adherence. These factors require an integrated approach by all professionals involved in the patient's care.

Adherence to therapy may be improved by any of a large number of interventions, which include influence the patient and mediation, but the key activity is a patient-provider partnership based on an informed respect for the patient's autonomy. This partnership, in turn, will serve as the foundation for other interventions aimed at improving adherence.
